# The effects of yoga on shoulder and spinal actions for women with breast cancer-related lymphoedema of the arm: A randomised controlled pilot study

**DOI:** 10.1186/s12906-016-1330-7

**Published:** 2016-09-02

**Authors:** Annette Loudon, Tony Barnett, Neil Piller, Maarten A. Immink, Denis Visentin, Andrew D. Williams

**Affiliations:** 1 Centre for Rural Health, University of Tasmania, Launceston, Tasmania 7250 Australia; 2 Department of Surgery, School of Medicine, Flinders University, Adelaide, South Australia 5042 Australia; 3 School of Health Sciences, University of South Australia, Adelaide, South Australia 5000 Australia; 4 School of Health Sciences, University of Tasmania, Locked Bag 1322, Launceston, TAS 7250 Australia

**Keywords:** Yoga, Arm lymphoedema, Breast cancer, Range of motion, Strength testing

## Abstract

**Background:**

We aimed to evaluate the effect of an 8-week yoga intervention on the shoulder and spinal actions of women with breast cancer-related arm lymphoedema.

**Method:**

A randomised controlled pilot trial. The intervention group (*n* = 12) completed eight weeks of daily yoga sessions while the control group (*n* = 11) continued with best current care including information on compression sleeves, skin care, risks of temperature variations and recommended safe use of affected arm. Lumbo-pelvic posture, range of motion (ROM) in the shoulder and spine, and strength in shoulder and pectoral major and minor, and serratus anterior were taken at baseline, week 8 and after a 4-week follow-up. Outcome assessors were blinded to allocation.

**Results:**

At week eight the intervention group had an improvement in lumbo-pelvic posture, as indicated by a reduction in pelvic obliquity compared to the control group (mean difference = −8.39°, 95 % CI: −15.64 to −1.13°, *p* = 0.023). A secondary finding was that strength in shoulder abduction significantly increased following the yoga intervention in both the affected (9.5 kg; CI: 0.34 to 18.66, *p* = 0.042) and non-affected arm (11.58 kg; CI: 0.25 to 22.91; *p* = 0.045). There were no significant between group changes in any ROM measures as a result of the yoga intervention.

**Conclusion:**

This pilot study demonstrates that participation in yoga may provide benefits for posture and strength in women with Breast Cancer Related Lymphoedema. The improvements may be attributed to the focus of yoga on overall postural and functional movement patterns. Further trials with longer intervention that follow this methodology are warranted.

**Trial registration:**

The Australian New Zealand Clinical Trials Registry ACTRN12611000202965.

## Background

In spite of improvements to surgery and radiation treatment, breast cancer-related lymphoedema (BCRL) continues to affect at least 20 % of women undergoing breast cancer treatment with axillary clearance [1]. As a chronic condition, lymphoedema requires life-long management to prevent the condition worsening and occurrence of infections such as cellulitis [2].

Upper body impairment is common in women with BCRL, whose reduction in functional activity can be higher than in women who have had breast cancer treatment without lymphoedema [3]. Impairment of the affected arm is evident in loss of shoulder range of motion (ROM) and strength [4] and changed bio-mechanics of the shoulder girdle, all leading to loss of symmetry between sides [5, 6]. These constraints produce difficulty with daily activities including household chores, driving, hobbies, carrying items and activities which require fine motor coordination of the hand [7]. Limitation of activity and level of pain have been associated with lower quality of life (QOL) [8]. Exercise interventions for women with BCRL have resulted in increased shoulder ROM [9], strength [10] and improved QOL [9], indicating that physical therapies may improve upper body morbidity [11].

Yoga is an integrated system that consists of breathing (pranayama), postures (asana), meditation and relaxation designed to improve the biopsychosocial functioning of an individual, irrespective of their limitations [12]. Improvements in physical flexibility, strength, neuro-motor coordination and postural alignment in a range of populations have been reported [13]. For this reason, yoga has been recommended as an adjunct to physiotherapy in a rehabilitation setting [14] including for those with lymphoedema [15] and may assist in correcting the upper body impairment frequently experienced by women with BCRL. However, currently the authors are unaware of any published research on the effect of yoga on posture, shoulder and spinal ROM and shoulder strength in women with BCRL.

The objective of this pilot trial was to obtain preliminary data to evaluate the effects of an 8-week yoga intervention on the upper body of women with stage one BCRL. This paper reports on outcomes of postural alignment, ROM of the shoulder and spine, and strength of the shoulder and other muscles affected by breast cancer surgery and treatment, i.e. pectoralis major, pectoralis minor and serratus anterior.

## Methods

### Study design

The trial was a multicentre parallel randomised controlled pilot trial (RCT) (Australian New Zealand Clinical Trials Registration ACTRN12611000202965). Ethics approval was granted by University of Tasmania Social Sciences Human Research Ethics Committee (H00011534).

The study was part of a larger study. The full protocol has been described previously [16] and the primary outcomes for the effects of yoga on lymphoedema and its sequelae and QOL have been published [17]. This manuscript presents the results for the secondary outcome measures of shoulder and spinal ROM and shoulder strength. Outcome measurements were performed at baseline, week 8 (on completion of intervention) and at week 12 (one month after intervention). Group allocation was conducted by a person independent of the trial using a computer-generated random number system on a 1:1 allocation ratio to intervention or control group. Notification of group allocation in sequentially numbered opaque sealed envelopes occurred after the baseline measurements. None of the assessors were involved in either the allocation or the delivery of the intervention. Participants were asked not to divulge their treatment to assessors and assessors did not have access to previous results.

#### Sample size calculation

A preliminary sample size calculation indicated that to detect a clinically significant increase in shoulder ROM of at least 10° between the two groups with expected within-group-differences of 10 % of the mean with 80 % power, so that 13–18 participants would be required for each group.

### Participants

Women were eligible for inclusion if they had unilateral secondary lymphoedema of the arm, stage one as defined by the International Society of Lymphology [18], confirmed by a professional lymphoedema therapist, had completed treatment for breast cancer (surgery, radiotherapy and chemotherapy) at least six months previously, were over 18 years and had sufficient English language comprehension to provide informed consent and understand yoga instruction. Women were advised that no new exercise should be commenced during the trial. Women were excluded if they had recurrent cancer, an infection or were having Complex Lymphoedema Therapy, if they were pregnant, wore a pacemaker, which would affect bioimpedance (BIS) readings, had severe psychological illness or were currently doing yoga. Women’s safety being paramount, it was thought that including only stage one BCRL may reduce the chance of a flare-up of lymphoedema occurring. The trial was advertised on local radio and in local newspapers, with lymphoedema therapists and breast care nurses throughout Tasmania and on community and health related noticeboards.

A manual containing information on best current care for BCRL was given to all participants at the commencement of the trial on the instructions of the overseeing ethics committee. Recommended current care included information on wearing of professionally fitted compression sleeve and when to wear it, continuing recommended skin care and avoiding cuts to the skin and extreme temperatures, wearing non-restrictive clothing, maintaining a healthy weight, and using the affected arm without undue strain such as repeating the same movement or holding the arm in one position for a long period of time. Women were advised to continue any current exercise, but not to commence any new activity during the trial period, and to seek immediate medical help in the event of an exacerbation of lymphoedema during the trial. Compliance to this request was measured using the International Physical Activity Questionnaire (IPAQ), a reliable measure of activity in this population [19] in the week prior to each measurement period. Daily physical activity was measured using a VAS scale as previously described [16].

### Control group

As lymphoedema is a medical condition that will worsen without treatment, participants randomised to the control group were requested to maintain their usual self-care throughout the trial period and were offered the same 8-week teacher led yoga with home-practice DVD as the intervention group at the completion of the final measurement.

### Yoga intervention group

The intervention consisted of a weekly 90-min teacher-led yoga class and a daily 45-min home-practice yoga session provided by DVD in addition to their usual self-care. Participants recorded their daily practice along with any relevant comments in a personal logbook. Women had the choice of wearing their compression sleeve during each session, as long as the compression sleeve was worn immediately on completion of each yoga session [20].

The Satyananda Yoga® style [21] was chosen as its systematised and progressive practices are based on an integrated system consisting of pranayama, asana, meditation and relaxation with options for modifications. The practices were chosen following the principles of manual lymphatic drainage [22], with gentle ROM actions of the shoulder and spine focussing on posture and kinematic movement patterns while engaging core and shoulder-stabilising muscles, and stress reduction. Safety precautions and exercise guidelines for women with BCRL were adhered to [23]. A full rationale [24] and basic outline of the yoga program [17] for the yoga group has been described previously.

### Outcome measurements

Anthropometric measurements were conducted and followed by shoulder ROM and strength tests with the same trained assessor at each time-point. A sub-group of women (*n* = 15), who volunteered for spinal ROM were tested on the day following other measurements. Participants were asked to volunteer separately for this assessment due to the need to partially undress for assessment, and were assured of the privacy they would be given.

### Range of motion of shoulder

Active shoulder ROM was measured according to an established reliable and validated method [25] using a two-armed goniometer [26]. Briefly, participants sat in a low-back chair with stable shoulder blades and suitable back support, their knees bent to 90° and their feet at hip width and flat on the floor. Flexion, abduction and extension of the shoulder in the sagittal or coronal planes were measured with the arms in anatomical position. Internal and external rotation of the shoulder were measured from the starting position of the arm abducted to 90°, forearm pronated and parallel to the floor, palm down, with elbow bent to 90°. To prevent fatigue of the affected arm, measuring was conducted in the following order: flexion, internal rotation, extension, abduction, external rotation. The endpoint of measurement was full range, compensatory movements of the shoulder or trunk occurring, or participant experiencing pain or tightness. The final score was recorded in degrees as the best of three attempts [25].

### Strength of shoulder and pectoralis major, pectoralis minor and serratus anterior

Muscle strength was measured according to an established protocol [27] using a reliable and valid handheld dynamometer (Commander Powertrack II Muscle Tester; JTechMedical, Salt Lake City, Utah, USA) [28]. The participant sat in a stable position and the non-affected, then the affected, arm was measured in turn, three times for each action. The arm was raised to 90° for the strength measurements of flexion, horizontal adduction and abduction. To measure the strength in extension, the participant’s arm was by her side. The arm was positioned slightly across the body for the strength measurement of the pectoralis major and the arm elevated to 120° for the serratus anterior. The strength of pectoralis minor was measured with the participant in supine position. This was also the order of measuring used to prevent fatigue.

The strength of the shoulder/separate muscles (pectoralis major, pectoralis minor, serratus anterior) was measured from the force applied against the resisted hand-held dynamometer, held by the assessor, for a count of three seconds. Measurement ceased when full strength was applied, compensatory movements of the shoulder or trunk occurred or pain was experienced. The best of three attempts was recorded in Newtons (N).

### Grip strength

Hand grip strength was assessed using a hand-held grip dynamometer (Smedleys, TTM, www.stoeltingco.com), validated for use in a clinical setting [29], as previously described [16].

### Spinal mobility

Spinal mobility was measured dynamically using video analysis in order to quantify the functional mobility of the spine during lateral flexion and flexion/extension from a stable standing position and thoracic spinal rotation in a stable sitting position according to established protocols [30]. Each movement was performed three times in a slow controlled fashion with no break between repetitions.

Movements were recorded by a video camera with backlighting, utilising reflective surface markers placed on participants’ skin at the following locations: left and right posterior superior iliac spines (LPSI, RPSI), spinal processes (S1, L3, L1, T6, T1) and left and right acromion (LACR, RACR). Reflective markers were also placed on the wall and floor for calibration. At the initial measurements, the distance between markers applied to the participant’s skin was recorded to ensure consistency.

Video data was analysed using Quintic™ Sports Biomechanics Video Analysis Software (9.03 version 14; Quintic Consultancy Limited; www.quintic.com). Measurements were made of the position of each marker in the reference plane from resting position to range in each direction. This allowed calculation of the angles in degrees defined in Table 1, following methods described elsewhere [30]. The range of excursion of each angle was recorded, except for pelvic obliquity and angle of kyphosis at rest which were static angles.

**Table 1:** Relationship between markers and angles for spinal mobility

Abbreviation	Action	Definition
Lateral Flexion		
β1	Pelvic obliquity at rest	Angle at rest between the pelvic axis (the vector LPSI-RPSI) and the horizontal plane.
β2	Lumbar lateral flexion range	Angle between S1-L1 vector and the vertical pelvic axis through full range of lateral flexion.
β3	Thoracic lateral flexion range	Angle between the T1-L1 vector and S1-L1 vector through full range of lateral flexion.
β4	Shoulder range	Angle between the vector connecting both shoulders with the horizontal plane through full range of lateral flexion.
βdc	Thoracic distal curvature range	Angle between vectors S1-L3 and L3-T6 through full range of lateral flexion.
βpc	Thoracic proximal curvature range	Angle between vectors L3-T6 and T6-T1 through full range of lateral flexion.
βlti	Full lateral trunk inclination range	Angle between the S1-T1 vector and the vertical axis through full range of lateral flexion.
Flexion-Extension/Rotation		
αkrest	Angle of thoracic kyphosis at rest	Angle at rest between the vectors L3 –T6 and T6-T1.
α3	Thoracic range	Angle between the vector T1-L1 and the vector S1-L1 through the full range of flexion/extension.
αfti	Full trunk flexion/extension range	Angle between the S1-T1 vector and the vertical axis in flexion/extension.
Tr	Thoracic spinal rotation range	Angle between the vector from the line of the shoulders (LACR – RACR) and the vector defined by the line of the hips (LPSI - RPSI) throughout the full range of thoracic spinal rotation.

### Data analysis

Baseline information for demographic and medical characteristics between treatment groups were compared by independent two-tailed t-tests for continuous variables and by Yates corrected chi-square tests for categorical variables (SPSS version 19; IBM, Armonk, New York, USA). Statistical analyses of outcome measures at baseline and changes between groups at weeks 8 and 12 were performed using STATA statistical software (version 12; STATA Corporation, College Station, Texas, USA). Parametric longitudinal data were analysed via mixed methods linear regression (ANOVA). Where assumptions of linear regression were violated, data were analysed using non-parametric analysis via ordinal logistic regression. Post-hoc testing was performed on all data using the Holms test to locate the means that were significantly different. Statistical significance was set at *p* < 0.05. Due to the low sample size neither multivariate nor covariate analyses were performed. Data is presented as Mean (M) and Standard Deviation (SD) unless otherwise indicated.

## Results

**Fig. 1:**
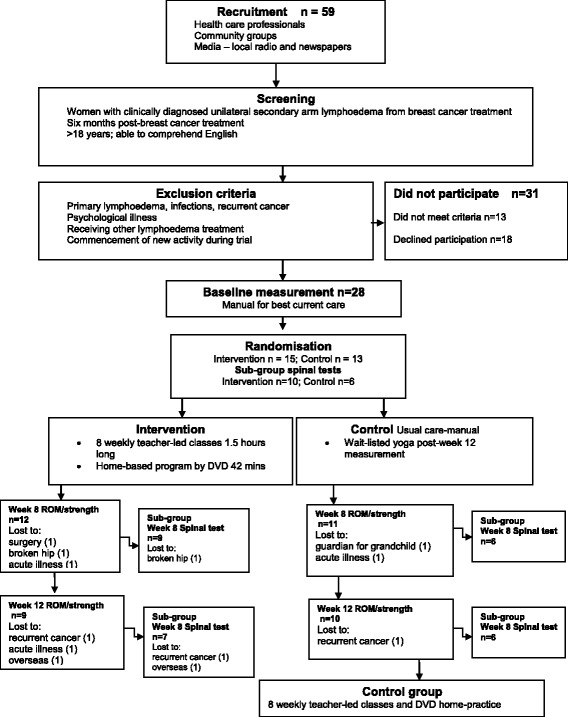
Flow of the trial

Participant flow through the trial is presented in Fig. 1. From 59 potential participants who expressed an interest in the study, 28 consented and underwent baseline testing with 15 randomised to the yoga intervention and 13 to the usual care control.

### Compliance with the intervention

Two participants withdrew after being diagnosed with recurrent cancer during the trial while seven others experienced adverse events requiring their withdrawal from the study that were unrelated to either their condition or the treatment. Briefly, five participants withdrew due to adverse events unrelated to the yoga intervention during the eight-week intervention period while another four withdrew between the end of the intervention and the follow up measurement (Fig. 1).

Due to these events, fewer participants returned for the week-12 follow-up than completed the week 8 measurements. Consequently, the results from baseline to end of the 8 week intervention (b-8) and end of the week 8 intervention to week 12 follow-up (8b-12) were analysed separately.

Apart from that prescribed for the intervention group, during the trial, no women in either group were doing yoga, nor had they done it since developing BCRL. Attendance at the yoga sessions was high (97 %), as was self-reported compliance with the home-practice DVD (86 %). There were no statistically significant changes in physical activity by either group across all time points as measured by either the IPAQ questionnaire or VAS scale.

### Characteristics of the group

There were no demographic or medical differences from diagnosis and treatment for breast cancer and lymphoedema in the groups at baseline (Table 2). Twenty-three participants, with a mean age of 57.6 ± 10.5 years (range 34–80) and a mean BMI of 27.2 ± 4.9 kg/m^2^ (range 20.4–37.3), completed the eight week intervention and 19 women returned for the week 12 follow-up measurement.

**Table 2:** Participants baseline and medical characteristics of the groups

Characteristic	Intervention (*n* = 12)	Control (*n* = 11)	*P* value^a^
Age (years; Mean ± SD)	55.1 ± 2.5	60.5 ± 3.6	0.230
Range	36–65	34–80	
Number nodes removed (Mean ± SD)	14.3 ± 2.6	11.2 ± 2.7	0.429
Range	5–30	2–25	
How long lymphoedema (years; Mean ± SD)	4.9 ± 1.6	5.5 ± 1.9	0.900
Range	6 months–20 years	1 month–23 years	
How long post-surgery (years; Mean ± SD)	1.2 ± 0.4	1.9 ± 0.7	0.822
Range	1 month–5 years	1 month–8 years	
	n %	n %	*P* value^b^
Occupation			
Home, retired	5 (42)	8 (73)	0.280
Employed	7 (58)	3 (27)	
Fitness (self-scored)			
Low	2 (17)	1 (9)	
Medium	8 (67)	8 (73)	0.913
High	2 (17)	2 (18)	
Most significant exercise			
Walking	8 (67)	8 (73)	0.890
Gardening	7 (58)	7 (64)	0.867
Gym - organised exercise	1 (8)	8 (73)	0.006
Type of surgery			
Lumpectomy	5 (42)	3 (27)	
Mastectomy	7 (58)	8 (73)	0.882
Type of lymph dissection			
Sentinel node	0	1 (9)	
Axillary clearance	12 (75)	10 (73)	0.980
Stage of breast cancer			
Ductal Carcinoma In Situ (DCIS)	0	1 (9)	
1	3 (25)	4 (36)	
2	6 (50)	5 (45)	0.976
3	3 (25)	1 (9)	
Treatment			
Chemotherapy	8 (66)	6 (54)	0.867
Radiotherapy	9 (75)	7 (64)	0.890
Most common area of radiotherapy			
Chest	7 (58)	7 (64)	
Axilla	2 (17)	2 (18)	0.909
Axilla and chest	3 (25)	2 (18)	
Lymphoedema dominant/non-dominant			
Dominant	7 (58)	7 (64)	0.867
Non-dominant	5 (42)	4 (36)	

*P < 0.05

^a^ *P* values obtained using two-tailed independent samples *t*-test

^b^ *P* values obtained using chi-square test with Yates correction

### Outcomes

#### BMI

At baseline, the intervention group had a higher BMI (Mean Difference (MD) 4.03 kg; 95 % Confidence Intervals (CI): 0.24 to 7.81; *p* = 0.023) than the control group. However, at week 8 (MD −0.6 kg; 95 % CI: −1.98 to −0.01; *p* = 0.147) and at week 12 (MD −0.41 kg; 95 % CI: −1 to 0.187; *p* = 0.378) this difference was not significant. Due to the low numbers in the trial no correlation analysis was carried out for the BMI baseline measurement.

#### Shoulder ROM

At baseline there was an inhibited ROM in all shoulder ROM measures across both groups with the affected arm exhibiting non-significant reduced ROM compared to the affected arm. There were no statistically significant differences in shoulder ROM between groups at baseline. From b-8, there was a difference in the change between groups in abduction (MD −14.70°; 95 % CI: −29.30 to −0.10; *p* = 0.049) and flexion (MD −19.00°; 95 % CI: −33.65 to −4.36; *p* = 0.011) of the non-affected arm due to the increase in abduction (MD 10.7°; 95 % CI:-0.08 to 21.48; *p* = 0.052) and flexion (MD 20.5°; 95 % CI: 9.68 to 31.32; *p* = 0.001) in the control group and little change in the intervention group (Table 3). Each group had significant changes for other actions (Table 3).

**Table 3:** Results shoulder ROM b-8 and 8b-12

Within group changes b-8				Between group changes b-8			Within group changes 8b-12			Between group changes 8b-12	
VariableGp (n)	Week 0 m ± SD	Week 8 m ± SD	P (0–8)	∆int-∆con 0–8 Md; (95 % CI)	P (0–8)	VariableGp (n)	Week 8b m ± SD	Week 12 m ± SD	P (8b)	∆int-∆con 8b-12 Md; (95 % CI)	P (8b-12)
Abduction affected											
Con(11)	91.82 ± 19.46	105.00 ± 14.01	0.008	−5.43;(−18.95 to 8.08)	0.431	Con(10)	104.40 ± 14.62	108.30 ± 16.49	0.344	−4.90; (−16.64 to 6.84)	0.413
Int(12)	88.17 ± 16.45	95.92 ± 15.70	0.104			Int(9)	92.56 ± 16.48	91.56 ± 10.25	0.818		
Abduction non-affected											
Con(10)	100.00 ± 18.45	110.70 ± 14.36	0.052	−14.70;(−29.30 to −0.10)	0.049	Con(9)	109.44 ± 14.64	114.22 ± 12.37	0.305	−3.00; (−15.92 to 9.92)	0.649
Int(12)	101.58 ± 17.75	97.58 ± 16.19	0.426			Int(9)	93.00 ± 16.17	94.78 ± 8.03	0.703		
Flexion affected											
Con(11)	101.82 ± 17.31	121.18 ± 16.89	0.001	−7.45;(−19.45 to 4.65)	0.228	Con(10)	119.80 ± 17.14	122.50 ± 16.96	0.530	−1.37; (13.61 to 10.87)	0.827
Int(12)	103.75 ± 12.47	115.67 ± 12.49	0.005			Int(9)	112.22 ± 12.62	113.56 ± 13.87	0.769		
Flexion non-affected											
Con(10)	106.10 ± 13.80	126.60 ± 13.37	0.001	−19.00;(−33.65 to −4.36)	0.011	Con(9)	125.33 ± 13.53	126.44 ± 16.68	0.847	4.89; (−11.12 to 20.89)	0.549
Int(12)	110.08 ± 10.23	111.58 ± 15.66	0.766			Int(9)	106.89 ± 15.26	112.89 ± 15.50	0.299		
Internal rotation affected											
Con(11)	47.18 ± 20.24	52.82 ± 18.76	0.526^a^	6.86;(7.20 to 20.92)	0.448^a^	Con(10)	52.70 ± 19.77	57.00 ± 19.43	0.056	−10.97;(−17.37 to −4.56)	0.001
Int(12)	45.83 ± 14.53	58.33 ± 11.51	0.050^a^			Int(9)	58.78 ± 11.44	52.11 ± 13.08	0.005		
Internal rotation non-affected											
Con(10)	47.40 ± 10.43	59.30 ± 13.12	0.031	−6.15;(−20.80 to 8.52)	0.411	Con(9)	59.22 ± 13.91	55.11 ± 15.07	0.316	1.67; (−9.71 to 13.04)	0.774
Int(12)	53.00 ± 17.49	58.75 ± 14.72	0.255			Int(9)	59.00 ± 15.98	56.56 ± 11.78	0.551		
Extension affected											
Con(11)	38.36 ± 9.29	48.27 ± 8.92	0.030	−0.41;(−9.41 to 8.59)	0.929	Con(10)	48.20 ± 9.40	51.60 ± 7.81	0.147	−5.96; (−12.64 to 0.73)	0.081
Int(12)	35.42 ± 11.45	44.92 ± 11.65	0.003			Int(9)	42.33 ± 12.21	39.78 ± 10.65	0.302		
Extension non-affected											
Con(10)	39.50 ± 9.25	48.30 ± 4.45	0.005^a^	3.03;(−6.12 to 12.18)	0.768^a^	Con(9)	47.89 ± 4.51	51.78 ± 7.26	0.120	−6.33; (−13.27 to 0.60)	0.073
Int (12)	37.42 ± 12.10	49.25 ± 11.69	0.021^a^			Int(9)	48.89 ± 13.37	46.44 ± 11.22	0.328		
External rotation affected											
Con(11)	65.54 ± 11.89	71.00 ± 6.29	0.041^a^	−2.20;(−12.34 to 7.95)	0.360^a^	Con(10)	70.30 ± 6.17	72.50 ± 6.65	0.282	−4.20; (−10.02 to 1.62)	0.157
Int(12)	63.33 ± 20.28	66.50 ± 11.12	0.945^a^			Int(9)	64.56 ± 12.20	62.56 ± 9.83	0.353		
External rotation non-affected											
Con(10)	70.50 ± 14.30	71.60 ± 14.20	0.702	−2.35;(−9.98 to 5.28)	0.546	Con(9)	70.89 ± 14.87	72.44 ± 13.69	0.353	0.89; (−3.76 to 5.53)	0.708
Int(12)	69.08 ± 12.32	67.83 ± 12.10	0.634			Int(9)	63.67 ± 10.28	66.11 ± 11.25	0.145		

∆ = change, *Gp* Group, *M ± SD* Mean ± Standard Deviation, *MD* Mean Difference, *CI* Confidence Interval, *n* number, *Con* control group, *Int* intervention group

^a^ non-parametric analysis

From 8b-12, the intervention group demonstrated decreased internal rotation of the affected arm compared to the control group (MD −10.97°; 95 % CI: −17.37 to −4.56; *p* = 0.001) due to the decrease in the intervention group (MD −6.67°; 95 % CI: −11.31 to −2.02; *p* = 0.005) and the increase in the control group (MD 4.3°; 95 % CI: −0.11 to 8.78; *p* = 0.056). Results for shoulder ROM are presented in Table 3.

#### Shoulder strength

There were no differences between groups in actions of shoulder strength at baseline. From b-8 and 8b-12, there were no significant changes between groups. However, from b-8, abduction of both the affected (MD 9.5 kg; 95 % CI: 0.34 to 18.66; *p* = 0.042) and the non-affected (MD 11.58 kg; 95 % CI: 0.25 to 22.91; *p* = 0.045) shoulder increased in the intervention group (Table 4).

**Table 4:** Results strength b-8 and 8b-12

Within group changes b-8		Between group changes b-8					Within group changes 8b-12			Between group changes 8b-12		
Variable Gp (n)	Week 0 m ± SD	Week 8 m ± SD	P (0–8)	P(0)	∆int-∆con 0–8 Md; (95 % CI)	P(0–8)	Variable Gp (n)	Week 8b m ± SD	Week 12 m ± SD	P (8b-12)	∆int-∆con 8b-12 Md; (95 % CI)	P(8b-12)
SHOULDER STRENGTH												
Abduction affected												
Con(11)	66.69 ± 27.75	71.11 ± 22.16	0.365	0.327	5.08; (−8.16 to 18.33)	0.452	Con(10)	71.18 ± 23.36	64.42 ± 21.17	0.074	2.89; (−7.88 to 13.67)	0.599
Int(12)	56.83 ± 23.26	66.33 ± 22.82	0.042				Int(9)	64.49 ± 23.54	60.62 ± 31.69	0.332		
Abduction non-affected												
Con(10)	71.82 ± 28.95	75.24 ± 21.77	0.573	0.246	8.16; (−8.26 to 24.58)	0.330	Con(9)	75.29 ± 23.09	70.09 ± 19.62	0.220	−1.20; (13.28 to 10.88)	0.846
Int(11)	59.18 ± 23.76	70.76 ± 26.07	0.045				Int(9)	68.75 ± 26.45	61.02 ± 22.61	0.153		
Flexion affected												
Con(11)	73.13 ± 24.33	78.51 ± 29.09	0.298	0.777	−2.83; (−16.86 to 11.20)	0.692	Con(10)	79.10 ± 30.60	76.04 ± 30.64	0.506	−0.56; (−13.67 to 12.54)	0.933
Int(12)	70.17 ± 24.20	72.72 ± 24.34	0.606				Int(9)	71.53 ± 26.62	67.91 ± 27.82	0.455		
Flexion non-affected												
Con(10)	69.04 ± 24.44	79.78 ± 28.66	0.071	0.964	−5.54; (−21.63 to 10.55)	0.500	Con(9)	81.07 ± 30.09	83.91 ± 30.38	0.525	−8.53; (−21.31 to 4.25)	0.191
Int(11)	68.53 ± 30.45	73.73 ± 25.17	0.359				Int(9)	74.98 ± 26.73	69.84 ± 31.86	0.230		
Extension affected												
Con(11)	93.44 ± 18.33	82.51 ± 31.07	0.098	0.293	−0.59; (−18.53 to 17.35)	0.949	Con(10)	83.06 ± 32.69	79.52 ± 26.76	0.504	−3.82; (−18.89 to 11.26)	0.620
Int(12)	81.85 ± 30.26	70.33 ± 29.12	0.069				Int(9)	64.51 ± 27.04	57.16 ± 25.52	0.187		
Extension non-affected												
Con (10)	88.72 ± 20.82	87.84 ± 29.56	0.902	0.329	−8.94; (−28.25 to 10.37)	0.364	Con(9)	86.60 ± 31.08	87.64 ± 32.16	0.833	−3.90; (−18.03 to 10.23)	0.588
Int (11)	78.58 ± 26.33	68.76 ± 20.62	0.149				Int(9)	67.33 ± 20.67	65.69 ± 28.97	0.585		
Horizontal adduction affected												
Con (11)	58.00 ± 19.86	60.80 ± 15.41	0.485	0.744	−1.88; (−12.77 to 9.00)	0.734	Con(10)	60.06 ± 16.04	58.52 ± 20.06	0.644	−4.82; (−14.32 to 4.69)	0.321
Int(12)	55.73 ± 18.56	56.65 ± 13.60	0.811				Int(9)	54.02 ± 13.21	47.67 ± 8.23	0.071		
Horizontal adduction non-affected												
Con(10)	59.60 ± 21.42	63.80 ± 13.44	0.356	0.851	−7.40; (−19.72 to 4.92)	0.239	Con(9)	63.56 ± 14.23	66.47 ± 18.32	0.376	−8.54; (−17.90 to 0.84)	0.074
Int(11)	58.20 ± 19.24	55.00 ± 14.82	0.461				Int(9)	53.90 ± 13.04	47.91 ± 9.12	0.105		
SEPARATE MUSCLES												
Pectoralis major affected												
Con(11)	53.99 ± 18.91	53.40 ± 14.36	0.891	0.918	−2.16; (−13.82 to 9.50)	0.717	Con(10)	52.80 ± 14.99	50.38 ± 11.52	0.327	−0.02; (−7.05 to 7.00)	0.995
Int(12)	54.82 ± 21.97	52.07 ± 17.05	0.504				Int(9)	50.60 ± 16.24	48.16 ± 13.85	0.347		
Pectoralis major non-affected												
Con(10)	55.00 ± 18.26	57.20 ± 14.67	0.549	0.679	−4.60; (−14.52 to 5.33)	0.364	Con(9)	54.76 ± 13.22	58.67 ± 15.12	0.133	−11.80; (−19.21 to −4.38)	0.002
Int(11)	58.40 ± 21.63	56.00 ± 19.04	0.493				Int(9)	54.45 ± 18.70	47.18 ± 15.01	0.004		
Pectoralis minor affected												
Con(11)	25.80 ± 6.89	28.80 ± 4.77	0.101	0.611	−0.98; (−5.95 to 3.98)	0.698	Con(10)	29.48 ± 4.42	22.44 ± 2.27	0.001	0.44; (−2.73 to 3.61)	0.786
Int(12)	26.95 ± 5.79	28.97 ± 4.48	0.250				Int(9)	29.33 ± 3.97	22.73 ± 4.92	0.001		
Pectoralis minor non-affected												
Con(11)	27.40 ± 8.93	29.20 ± 6.82	0.495	0.898	−0.80; (−8.11 to 6.51)	0.830	Con(10)	29.26 ± 7.19	22.22 ± 5.33	0.002	1.46; (−5.11 to 8.03)	0.663
Int(11)	27.00 ± 8.81	28.00 ± 7.24	0.705				Int(9)	28.60 ± 7.98	22.98 ± 4.68	0.024		
Serratus Anterior affected												
Con(11)	37.77 ± 7.02	46.00 ± 15.16	0.059	0.657	1.09; (−9.93 to 12.10)	0.847	Con(10)	45.32 ± 15.80	46.20 ± 16.17	0.168^a^	−3.32; (−13.91 to 7.26)	0.264^a^
Int(12)	35.86 ± 13.88	44.18 ± 12.18	0.008				Int(9)	47.44 ± 10.58	44.00 ± 10.08	0.593^a^		
Serratus Anterior non-affected												
Con(10)	33.00 ± 7.89	49.94 ± 13.24	0.001	0.981	−4.28; (−11.46 to 2.90)	0.243	Con(9)	50.36 ± 13.98	45.78 ± 15.59	0.190	1.35; (−8.58 to 11.29)	0.789
Int(11)	37.18 ± 12.29	45.80 ± 10.68	0.001				Int(9)	45.93 ± 9.14	43.27 ± 7.04	0.379		
Handgrip affected												
Con(11)	24.6 ± 5.9	25.5 ± 3.9	0.439	0.866	−0.905 (−3.935 to 2.125)	0.558	Con(10)	24.8 ± 3.47	21.55 ± 3.72	0.002	3.58; (1.50 to 5.67)	0.01^a^
Int (12)	25.1 ± 8.9	25.0 ± 7.4	0.969				Int(9)	24.39 ± 7.70	24.72 ± 8.72	0.525^a^		
Handgrip non affected												
Con (11)	25.5 ± 3.5	25.3 ± 3.9	0.768	0.481	1.583 (−0.666 to 3.833)	0.168	Con(9)	24.39 ± 2.95	24.06 ± 2.18	0.672	0.83; (−1.35 to 3.02)	0.455
Int (12)	23.8 ± 7.1	25.1 ± 6.9	0.085				Int(9)	23.22 ± 6.36	23.72 ± 7.04	0.526		

Baseline (b) = 0

∆ = change, *Gp* Group, *M* ± *SD* Mean ± Standard Deviation, *MD* Mean Difference, *CI* Confidence Interval, *n* number, *Con* control group, *Int* intervention group

^a^ non-parametric analysis

#### Strength of pectoralis major, pectoralis minor and serratus anterior

There were no differences between groups in strength actions for these muscles at baseline. From b-8, there were no changes between groups. From 8b-12, there was a decrease in the strength of the non-affected pectoralis major in the intervention compared to the control group (MD −11.80 N; 95 % CI: −19.21 to −4.38; *p* = 0.002) due to the decrease in the intervention group (MD −7.88 N; 95%CI −13.27 to −2.50; *p* = 0.004). Both groups had significant increases for serratus anterior at week 8 and significant decreases for pectoralis minor at week 12 (Table 4).

#### Grip strength

There were no differences between groups in grip strength at baseline or from b-8. From 8b-12 there was a decrease in grip strength of the affected arm (MD 3.58 kg; 95 % CI:1.50 to 5.67; *p* = 0.01) due to the decrease in the control group (MD −3.25 kg; 95 % CI −4.69 to −1.82; *p* = 0.002) (Table 4).

#### Spinal mobility

At baseline, the intervention group had a higher (worse) angle of pelvic obliquity compared to the control group (MD +9.97°; 95 % CI: 2.76 to 17.17; *p* = 0.007). From b-8, the angle of pelvic obliquity was lower (improved) in the intervention group compared to the control group (MD −8.39°; 95 % CI: −15.64 to −1.13; *p* = 0.023) due to the reduction in pelvic obliquity in the intervention group (MD −9.96°; CI: −14.54 to −5.37; *p* = 0.001).

At baseline, the angle of thoracic kyphosis at rest was higher (worse) in the intervention group (MD 8.13°; 95 % CI: −0.10 to 16.37; *p* = 0.053). From b-8, there was a tendency towards reduction (improvement) for the angle of kyphosis in the intervention group compared to the control group (MD −3.88°; 95 % CI −8.08 to 0.32; *p* = 0.070).

No between-group changes from b-8 or 8b-12 were observed for any other measure of spinal mobility (Table 5).

**Table 5:** Results spinal mobility b-8 and 8b-12

Within group changes b-8				Between group changes b-8			Within group changes 8b-12			Between group changes 8b-12		
VariableGp (n)	Week 0 m ± SD	Week 8 m ± SD	P (0–8)	P (0)	∆int-∆con 0–8 Md; (95 % CI)	P (0–8)	Variable Gp (n)	Week 8b m ± SD	Week 12 m ± SD	P (8b-12)	∆int-∆con 8b-12 Md; (95 % CI)	P (8b-12)
LATERAL FLEXION												
Pelvic Obliquity β1												
Con(6)	15.40 ± 6.23	13.83 ± 6.09	0.585	0.007	−8.39; (−15.64 to −1.13)	0.023	Con(6)	13.83 ± 6.09	13.72 ± 8.79	0.972	0.52; (−8.29 to 9.33)	0.908
Int(9)	25.37 ± 6.90	15.41 ± 7.94	0.001				Int(7)	13.44 ± 5.58	13.84 ± 7.22	0.896		
Lumbar range β2												
Con(6)	24.82 ± 15.37	17.27 ± 9.12	0.016	0.521	6.48; (−1.43 to 14.40)	0.109	Con(6)	17.27 ± 9.12	19.33 ± 6.11	0.512	−5.54; (−13.96 to 2.88)	0.197
Int(9)	21.18 ± 10.35	20.11 ± 8.34	0.676				Int(7)	20.56 ± 9.12	17.09 ± 4.53	0.234		
Thoracic range β3												
Con(6)	48.80 ± 11.71	51.90 ± 3.14	0.286	0.543	0.72; (−6.63 to 8.08)	0.847	Con(6)	51.90 ± 3.14	55.63 ± 6.16	0.103	−2.91; (−9.02 to 3.21)	0.352
Int(9)	45.18 ± 14.55	49.00 ± 10.52	0.107				Int(7)	48.86 ± 9.31	49.69 ± 13.22	0.696		
Shoulder range β4												
Con(6)	92.08 ± 17.91	86.55 ± 11.96	0.151	0.962	3.21; (−6.55 to 12.97)	0.519	Con(6)	86.55 ± 11.96	87.77 ± 13.56	0.730	−2.06; (−11.47 to 7.35)	0.668
Int(9)	92.64 ± 25.78	90.32 ± 26.22	0.461				Int(7)	86.24 ± 26.01	85.40 ± 23.43	0.796		
Thoracic distal curvature range βdc												
Con(6)	47.83 ± 12.60	48.40 ± 6.16	0.920	0.518	6.50; (−7.70 to 20.70)	0.370	Con(6)	48.40 ± 6.16	43.42 ± 8.16	0.102	4.44; (−3.70 to 12.58)	0.285
Int(9)	43.72 ± 11.78	50.79 ± 14.59	0.123				Int(7)	50.99 ± 16.75	50.44 ± 17.53	0.847		
Thoracic proximal curvature range βpc												
Con(6)	29.62 ± 14.55	31.65 ± 9.83	0.663	0.898	−5.52; (−17.34 to 6.30)	0.360	Con(6)	31.65 ± 9.83	34.35 ± 13.48	0.408	−0.54; (−9.26 to 8.18)	0.903
Int(9)	30.66 ± 20.07	27.17 ± 13.23	0.360				Int(7)	26.20 ± 11.47	28.36 ± 10.77	0.475		
Full lateral trunk inclination range βlti												
Con(6)	60.12 ± 13.61	52.70 ± 7.60	0.024	0.505	−0.87; (−9.19 to 7.45)	0.837	Con(6)	52.70 ± 7.60	55.22 ± 10.16	0.406	−2.87; (−10.97 to 5.22)	0.486
Int(9)	65.07 ± 16.45	56.78 ± 14.88	0.002				Int(7)	55.47 ± 15.58	55.11 ± 12.13	0.899		
FLEXION EXTENSION/ROTATION												
Angle of thoracic kyphosis at rest αkrest												
Con (6)	23.47 ± 8.68	25.80 ± 5.72	0.160	0.053	−3.88; (−8.08 to 0.32)	0.070	Con(6)	25.80 ± 5.72	21.57 ± 9.18	0.039	2.48; (−3.01 to 7.96)	0.376
Int (9)	31.60 ± 10.45	30.06 ± 7.86	0.254				Int(7)	30.69 ± 8.68	28.93 ± 10.85	0.355		
Thoracic range α3												
Con (6)	58.58 ± 10.03	58.63 ± 10.06	0.995	0.053	5.34; (−15.22 to 25.90)	0.611	Con(6)	58.63 ± 10.06	57.52 ± 14.28	0.873	3.95; (−14.73 to 22.62)	0.679
Int(9)	43.40 ± 16.90	48.79 ± 17.12	0.417				Int(7)	54.57 ± 14.38	57.40 ± 13.00	0.662		
Full trunk flexion/extension range αfti												
Con(6)	163.78 ± 20.43	175.02 ± 1.88	0.016^a^	.098^a^	7.48; (−31.00 to 45.95)	0.684^a^	Con(6)	175.02 ± 1.88	166.88 ± 19.33	0.168	7.08; (−8.66 to 22.82)	0.378
Int(9)	135.93 ± 38.86	154.64 ± 30.64	0.125^a^				Int(7)	151.56 ± 34.21	150.50 ± 39.95	0.846		
Thoracic spinal rotation range Tr												
Con(6)	84.23 ± 28.98	87.00 ± 20.01	0.764	0.171	8.28; (−15.05 to 31.60)	0.487	Con(6)	87.00 ± 20.01	93.62 ± 22.60	0.141	−7.79; (−19.79 to 4.21)	0.203
Int(9)	66.22 ± 28.33	77.27 ± 21.07	0.142				Int(7)	77.06 ± 13.80	75.88 ± 16.14	0.778		

Baseline(b) = 0

∆ = change, *Gp* Group, *M* ± *SD* Mean ± Standard Deviation, *MD* Mean Difference, *CI* Confidence Interval, *n* number, *Con* control group, *Int* intervention group

^a^ non-parametric analysis

## Discussion

To the best of our knowledge this was the first study to examine the effects of a yoga intervention on shoulder and spinal ROM and shoulder strength in women with BCRL. The major finding was that pelvic obliquity was reduced following the eight week yoga intervention. A secondary finding was that strength was increased following the yoga intervention for shoulder abduction in both the affected and non-affected arm.

There was noticeable lateral tilt of the pelvis (as measured by pelvic obliquity) for both the control and intervention group at baseline indicating variation from ideal posture. Changes in posture may be affected by a number of factors for this cohort including complications associated with BCRL. The one study on body posture of those with BCRL [5] reported that participants displayed mediolateral pelvic movement and dropped shoulder to the affected side when walking. In addition faulty body posture has been reported after breast cancer treatment of mastectomy [31] a high risk factor for BCRL, indicating a forward leaning of the trunk and lack of symmetry in the trunk and shoulders. These changes may be a result of the swelling, the different weight distribution of the lymphoedematous limb and changed biomechanics of the trunk and shoulder girdle from breast cancer treatment, similar to what has been reported for those with lower limb lymphoedema [32]. Nevertheless it must be acknowledged that it is not uncommon for women in this age group (median age 58) to have pelvic obliquity from various age and life related stresses on the lower body and pelvis including the effects of childbirth [33] affecting postural alignment, pelvic stability, and walking gait and hence the observed pelvic obliquity may not be solely associated with BCRL.

The yoga intervention resulted in an improvement (reduction) in a static postural measure, the angle of pelvic obliquity, between the groups (*p* = 0.023) due to an improvement in the yoga intervention group (*p* = 0.001). In addition thoracic kyphosis, demonstrated a non-significant improvement for the yoga group compared to the control group (*p* = 0.07). While the intervention group was outside the normative values for this measure at baseline [30], there were no significant changes in either group. While the differences between the groups at baseline limit the ability to form conclusions about the changes observed for this measure, changes in the angle of kyphosis may need a longer intervention than the current trial with reductions in kyphosis in one yoga trial reported following three one hour yoga sessions per week for 24 weeks [34]. Yoga focusses on teaching postural alignment from the feet through to the pelvis, shoulders, neck and head before commencing any of the physical, breathing or meditative practices. There is also a strong focus on body awareness (*kaya sthairyam*). In the current trial postural alignment and awareness were taught before, during and after each practice in order to create a stable foundation. Core stabilisation is also a key feature of yoga and is achieved by engaging the muscles of the transversus abdominis and pelvic floor (*moolabhanda*) before engaging other muscles in many yoga postures or as part of breathing practices. While core strength was not measured in the current trial, the improvement in pelvic obliquity indicated at least an awareness of maintaining pelvic stability [15]. Pelvic stability while standing was improved following core stability exercises [35] and in a Pilates intervention [36]. Although there were improvements in pelvic stability in the intervention group it must be acknowledged that differences existing at baseline meant that the potential for improvement was high.

Strength in shoulder abduction improved in both arms following the yoga intervention. However, these were not significantly different to changes observed following the control period, possibly due to the small sample size in this pilot study. Nevertheless these results support previous uncontrolled studies showing improvements in handgrip strength following a yoga intervention [37] although this was not supported by any improvements in hand grip strength in the current study. The yoga intervention had no weight-bearing on the arms; however, the focus on stabilisation of the scapula/shoulder complex (*amsa bandha*,) occurred for every action. Correct scapula/shoulder stabilisation is dependent on space in the sub-acromial area and scapulohumeral rhythm relies on the correct firing of key stabilising muscles such as the upper and lower trapezius, serratus anterior, pectoral muscles and the rhomboids [38], which can be adversely affected by breast cancer treatment [39]. Shoulder abduction requires contraction of the supraspinatus and deltoid muscles, which occurs in a fluid action when this sub-acromial space exists. In the current trial, the focus on shoulder stability may have led to improved scapulohumeral rhythm facilitating abduction strength. Another trial based on gentle exercises, similar to the yoga intervention, to achieve scapulohumeral control in a group of participants with scapular dyskinesis (*n* = 18) showed that specific gentle shoulder movements activated the upper and lower trapezius and serratus anterior in both groups [40]. We have previously reported reduced symptoms and tissue density in the upper arm and reduction in fluid of the affected arm for the intervention group [17]. It is likely that these improvements made the action of abduction easier in the same way reduction of fluid and improved postural alignment and stability improved walking gait of those with lower limb lymhoedema [32].

The intervention was not associated with increases in ROM across any shoulder movement. The focus on creating stability of the glenohumeral complex may explain the lack of increase in ROM. Similar studies involving shoulder stability [41] or Pilates [36] also found no changes in shoulder ROM. Interestingly, the Pilates intervention reported improved biomechanical control of the scapula and trunk in lateral flexion [36], perhaps indicating that stability is achieved prior to changes in ROM. It is possible that the ROM results are due to a learning effect from baseline to week eight as there was a mean increase in 18 of the 20 measures of shoulder ROM in both the intervention and control group over this timeframe. The fact that this pattern did not continue from week 8 to 12 gives weight to this hypothesis. While further study is required to determine the true effects of a yoga intervention on ROM in this population the results highlight the potential importance of inclusion of a familiarity phase prior to data collection in any future study.

There were no significant between group changes in spinal ROM. In comparison to norms for spinal mobility [30], group mean baseline measurements revealed participants in the current trial recorded lower flexion/extension and lateral flexion although they were within the normal range for rotation. However, there was no change in spinal mobility as a result of the yoga intervention. Similar findings were observed following 12 weeks of Pilates [36] where it was suggested that participants reduced compensatory movements of the spine by focussing on segmental motion, postural alignment and stability rather than on increasing their range. The results of the current trial support this hypothesis.

As already acknowledged there are several limitations in this study. These include the lack of familiarity testing prior to data collection, the differences between the groups in certain variables at baseline, as well as the small sample size. Due to a lack of prior data to use to calculate sample size, the a priori sample size calculation performed for this study was based on clinically significant changes in the major outcome variables with an assumed level of variability [16]. This indicated that between 13–19 participants would be required per group and we proposed that 20 participants per group would be recruited. Unfortunately we did not achieve the proposed sample size and there was greater variability in the results than anticipated resulting in wider confidence intervals than expected. These factors impacted on the statistical significance of results including potentially the differences between the groups at baseline. Nevertheless the pilot study results obtained provide important initial data which will inform sample size calculations for any future studies.

## Conclusions

This pilot study highlights possible improvements in posture evident in the reduction in the angle of pelvic obliquity and trend to a reduced angle of kyphosis as a result of the yoga intervention. Further, the intervention group increased shoulder strength for both arms in the action of abduction, an indicator that improved shoulder stability may have occurred. The improvements observed in this study may possibly be attributed to the focus of the yoga intervention on overall placement of body posture and function in each aspect of the yoga during meditation, breathing, relaxation and execution of the physical postures both moving and stationary. Such increased awareness in body placement and method of movement rather than a focus on increasing flexibility of movement may account for the lack of change in shoulder and spinal ROM. As women with BCRL can experience problems with posture and shoulder actions these results possibly indicate that yoga may offer some positive outcomes. While there are several limitations including a small sample size, the data provides sufficient evidence of the potential benefits of yoga to warrant larger controlled trials following the general methodology outlined in this paper.

## Abbreviations

BCRL: Breast cancer related lymphoedema
ROM: Range of motion
QOL: Quality of life
RCT: Randomised controlled trial
BIS: Bioimpedance spectroscopy
LPSI: Left posterior superior iliac spine
RPSI: Right posterior superior iliac spine
LACR: Left acromion
RACR: Right acromion
